# Reply: “Human Pose Estimation Is Approximate: Limitations and Considerations for its Use in Clinical Trials”

**DOI:** 10.1002/mdc3.70075

**Published:** 2025-04-12

**Authors:** Carlo Alberto Artusi, Christian Geroin, Serena Camozzi, Stefano Aldegheri, Nicola Bombieri

**Affiliations:** ^1^ Department of Neuroscience “Rita Levi Montalcini” University of Turin Turin Italy; ^2^ Neurology 2 Unit A.O.U. Città della Salute e della Scienza di Torino Torino Italy; ^3^ Department of Surgery, Dentistry, Paediatrics and Gynaecology University of Verona Verona Italy; ^4^ Neurology Unit, Movement Disorders Division, Department of Neurosciences Biomedicine and Movement Sciences University of Verona Verona Italy; ^5^ Department of Computer Science University of Verona Verona Italy

**Keywords:** postural abnormalities, pisa syndrome, camptocormia, Parkinson's disease, quantitative assessment

We thank Dr. Kondo for the comments reported in his letter regarding our study on AutoPosturePD.^1^, ^2^ We appreciate his perspective and acknowledge that the gold standard for identifying anatomical landmarks of spinal levels is instrumental, involving imaging methods such as X‐ray, magnetic resonance imaging, or computed tomography. To date, direct measurement on the patient's back by palpation‐based identification of anatomical landmarks, even when performed by trained medical professionals, is practical but has demonstrated limited accuracy.^3^ Therefore, while the spinal level can be identified using one or more of these modalities, the major advantage of AutoPosturePD, based on the Human Pose Estimation (HPE), lies in its ability to automatically detect the angles of axial postural abnormalities according to the recently proposed measurement methods and diagnostic criteria.^4^

In our previous study,^5^ we validated AutoPosturePD against the “gold standard” (manual measurement), demonstrating low margins of error, with intraclass correlation coefficients above 0.9 and a mean difference compared to the anatomical landmark assessment of 0.6 ± 3.1° for lateral trunk flexion, 0.3 ± 2.5° for anterior trunk flexion with thoracic fulcrum, and 1.3 ± 1.8° for anterior trunk flexion with lumbar fulcrum.^5^ Because such validation confirms the high accuracy of AutoPosturePD relative to the gold standard (assuming in‐person anatomical landmark assessment is considered the gold standard for this type of evaluation), we see no reason why it should not be considered a viable outcome measure in future clinical trials. Of course, this should ideally be preceded by further validation in a larger PD population, as well as external validation by independent groups using different datasets.

In fact, while it is true that AutoPosturePD measurements are derived from computational models,^6^ this is also the case for many widely accepted clinical tools. Many commonly used “derived” measures, such as gait analysis with wearable inertial sensors, are now being incorporated into clinical trials after validation against gold‐standard counterparts (eg, infrared motion capture).

AutoPosturePD's primary advantage in assessing axial postural abnormalities in PD lies in its ability to provide a time‐efficient, automated measurement that can be performed remotely. This is particularly relevant in clinical and research settings where in‐person evaluations may not always be feasible.

Over the past decade, technological advancements in Deep Learning applications for video analysis have significantly improved the accuracy of human keypoint prediction, leading to the widespread adoption of HPE software for human motion analysis. Beyond traditional application domains such as sports performance analysis, human‐robot interaction, and gesture recognition, numerous research studies have implemented this computer vision technology for clinical applications. A common perspective across these studies is that motion analysis using HPE software represents a very near future in trials and clinical practice.

In conclusion, we appreciate the discussion on this topic and believe that, as technology evolves, automated tools like AutoPosturePD can complement traditional assessments, ultimately enhancing accessibility and efficiency in both clinical practice and research settings.

## Author Roles

All authors have participated in the article preparation. All authors have approved the final submitted article. This article represents original work by the authors, has not been published elsewhere, and is not under consideration for publication elsewhere. (1) Research Project: A. Conception, B. Organization, C. Execution; (2) Statistical Analysis: A. Design, B. Execution, C. Review and Critique; (3) Manuscript Preparation: A. Writing of the first draft, B. Review and Critique.

C.A.A.: 1A, 1C, 3A, 3B

C.G.: 1B, 1C, 3A, 3B

S.C.: SA: 1C, 3B

S.A.: 1C, 3B

N.B.: 1A, 1B, 3B

## Disclosures

**Ethical Compliance Statement:** The authors confirm that the approval of an institutional review board was not required for this work. Informed consent was not obtained as no patients were involved. We confirm that we have read the Journal's position on issues involved in ethical publication and affirm that this work is consistent with those guidelines.

**Funding Sources and Conflict of Interest:** None.

**Financial Disclosures for the Previous 12 Months:** CAA received speaker honoraria from Abbvie, Bial, Zambon, Lusofarmaco. CG reports no financial disclosures. SC reports no financial disclosures. SA reports no financial disclosures. NB reports no financial disclosures. The authors declare that there are no additional disclosures to report.

## Data Availability

No data to be shared.
